# An Emerging New Paradigm in Opioid Withdrawal: A Critical Role for Glia-Neuron Signaling in the Periaqueductal Gray

**DOI:** 10.1100/2012/940613

**Published:** 2012-08-02

**Authors:** Handong Ouyang, Shue Liu, Weian Zeng, Roy C. Levitt, Keith A. Candiotti, Shuanglin Hao

**Affiliations:** ^1^Department of Neurology, University of Michigan Medical School, Ann Arbor, MI 48109, USA; ^2^Department of Anesthesiology, University of Miami Miller School of Medicine, Miami, FL 33136, USA; ^3^Department of Anesthesiology, State Key Laboratory of Oncology on Southern China, Cancer Center, Sun Yat-Sen University, Guangzhou 510060, China

## Abstract

The chronic use of opiates (i.e., narcotics such as the natural derivatives of opium including morphine or codeine) or opioids (i.e., semisynthetic derivatives of opium and other molecules that activate opioid receptors) induces dependence, which is associated with various specific behavioral and somatic signs after their withdrawal or after the administration of an opioid antagonist. Among the brain regions implicated in opiate dependence and withdrawal, the periaqueductal gray area (PAG) appears to be critical in regulating the complex signs and symptoms of opioid withdrawal. Numerous neurochemical mechanisms in the PAG have been identified that may contribute to the opioid withdrawal syndrome. Accumulating evidence suggests that glial activation leading to the release of proinflammatory molecules acting on neurons is important in the complex syndrome of opioid dependence and withdrawal. This paper focuses on the recent advances in our understanding of the vital role that glia-neuron interactions play in opioid dependence and withdrawal within the PAG. We summarize those neurochemical mechanisms associated with opioid withdrawal including the recently defined importance of TNF**α** release from activated glial cells that communicate with TNF receptors on PAG neurons.

## 1. Introduction

Addiction to the illicit and prescribed use of opiate narcotics is a significant public health issue [1]. In the United States, an estimated 22.6 million Americans aged 12 or older were illicit drug users (Report from the 2012 National Survey on Drug Use and Health: National Findings. Substance Abuse and Mental Health Services Administration, Department of Health and Human Services). Addiction to opioids is a complex syndrome involving tolerance, drug-seeking, and physical dependence with withdrawal avoidance behaviors [2]. Physical withdrawal is a major cause of compulsive drug-taking behavior and short-term relapse [3, 4]. The severity of opioid dependence and the somatic symptoms triggered by withdrawal are major contributors to the addictive potential of opioid narcotics (Nestler EJ, Mount Sinai Medical Center, personal communication). Chronic opioid use in patients is further complicated by increased drug requirement for efficacy as well as hyperalgesia. Opioid withdrawal is believed to result from adaptations on multiple levels within the nervous system. Functional studies have implicated an important role for the periaqueductal gray (PAG) in the expression of many signs and symptoms of opioid withdrawal, but the cellular and molecular mechanisms are not fully understood. While opioid receptor-effector uncoupling has been implicated in physical dependence, this phenomenon cannot fully account for withdrawal signs and symptoms or rebound responses in neurons after the administration of opioid receptor antagonists [5]. Recently, the importance of glial activation and the release of proinflammatory cytokines within the PAG acting on neuronal receptor in withdrawal responses has been reported [6]. This topical report will review these recent findings and this emerging paradigm whereby these phenomena maybe mechanistically linked and better explain the complex syndrome of opioid dependence and withdrawal.

## 2. Opioid Withdrawal in Humans and Animal Model Systems

The term dependence will be used here in the context of the withdrawal syndrome that is characteristically observed upon the cessation of opioids after chronic use, or associated with the administration of opioid antagonists. In humans, the signs and symptoms of withdrawal include stomach cramps, diarrhea, rhinorrhea, sweating, elevated heart rate and increased blood pressure, irritability, dysphoria, hyperalgesia, and insomnia [7, 8]. After abrupt cessation of heroin or morphine use, the withdrawal syndrome develops within 24 hours and generally persists with declining severity for 1 week to 10 days. However, dysphoria and anhedonia can persist for much longer [8]. The withdrawal syndrome contributes to opioid addiction during cycles of opioid use or abuse presumably because repeated dosing is maintained or escalated to avoid the withdrawal syndrome leading to development of more profound tolerance and dependence [2, 8]. Furthermore, naloxone-precipitated withdrawal has been used to quantify opioid dependence in human volunteers [9, 10].

These signs and symptoms found in humans can be replicated in models of compulsive drug use in animals [8]. As such, animal models have very strong predictive validity with regard to human dependence for the same opioids [8]. For example, these signs are readily observed and quantified following administration of antagonists such as naloxone (termed “naloxone-precipitated withdrawal”) or after abrupt cessation of treatment with relatively short-acting opioids (termed “spontaneous withdrawal”) [8]. In rats and mice, sympathetic and parasympathetic imbalances associated with signs of opioid withdrawal include, but are not limited to, “wet-dog” shakes, jumps, burrowing, hyper-reactivity, abnormal posture, teeth chatter, piloerection, ptosis, lacrimation, rhinorrhea, diarrhea, abrupt weight loss, penile erection, and ejaculation [8, 11, 12]. In these models, the brain regions contributing to the physical signs of opiate withdrawal include the periaqueductal gray (PAG) area, the locus coeruleus, amygdala, ventral tegmental area, nucleus accumbens, hypothalamus, and spinal cord [13]. 

## 3. Anatomical Overview of the PAG: Afferents and Efferents 

The PAG constitutes a cell-dense region, bordered laterally by the descending tectospinal fibers, which surrounds the midbrain aqueduct [14]. In the rostral midbrain (at the level of posterior commissure) as the third ventricle narrows to become the midbrain aqueduct, the PAG forms as an elongated, oval-shaped collection of neurons in continuity with the periventricular gray matter of the hypothalamus [14]. The functional studies provided a basis to subdivide the PAG into dorsal and ventrolateral longitudinal columns [14]. Both the ventrolateral PAG (vlPAG) and lateral PAG (lPAG) project extensively to ventromedial and ventrolateral medullary regions [14]. Distinct PAG columns project to specific hypothalamic and midline and intralaminar thalamic regions [15, 16]. The vlPAG receives afferents from the dorsal horn of the spinal cord and the nucleus of the solitary tract and projects to both the rostral and caudal ventrolateral medulla and the ventromedial medulla [14]. Amygdaloid projections to the PAG arise principally from the central nucleus and terminate in all but the dlPAG column. The lateral hypothalamic area projects selectively to the vlPAG [14]. Many of these nuclei are associated with withdrawal behaviors [17].

## 4. The Role of the PAG in Morphine Withdrawal

Functional studies have implicated a role for the PAG in the expression of many signs of opioid withdrawal, but the molecular mechanisms are not fully understood. The anatomical sites that mediate the diverse symptoms of physical opiate withdrawal have been explored using both intracerebral injections of opioid receptor antagonists and central nervous system lesions in dependent animals [11]. The PAG is rich in opioid receptors and endogenous opioid peptides and mediates physiological functions [17, 18]. Opioid antagonists microinjected into the PAG elicit strong withdrawal signs in rats implanted with pellets of morphine [11, 19]. Intraperitoneal naloxone precipitates morphine withdrawal signs in rats with chronic morphine infused into the PAG [20]. Endogenous peptide *β*-endorphin infused into the PAG for 72 h followed by naloxone elicits a typical morphine withdrawal-like syndrome [21]. The number of Fos immunoreactive neurons in the lateral and ventrolateral subdivisions of the PAG is increased after opioid withdrawal in both awake and anesthetized rats, which is most predominant in the caudal areas of the ventrolateral PAG [22–24]. Electrophysiologic studies during opioid withdrawal on opioid-sensitive neurons in the PAG display enhanced activity caused by induction of a novel opioid-sensitive currents distinct from the potassium conductance modulated by acute exposure to opioids [25]. Withdrawal induced neuronal activation occurs in lateral and ventrolateral columns of neurons, and particularly the caudal ventrolateral PAG [23]. Taken together, the data suggest that the PAG performs a key role in the phenomena of morphine withdrawal. 

## 5. Neurochemical Changes within the PAG Associated with Morphine Withdrawal

Many attempts have been made to investigate neuronal mechanisms involved in opioid dependence and withdrawal responses. A large range of neurochemical mechanisms have been identified in the neurons that may contribute to the opioid withdrawal syndrome, some of which are highlighted here.

### 5.1. Enkephalins and the Neuroanatomical Localization of Opioid Withdrawal

The PAG in the adult rodent brain contains a large number of opioid receptors and their naturally occurring peptide ligands [18] and is therefore very sensitive to the administration of opioid receptor antagonists and agonists [13]. Preproenkephalin (PPE) mRNA in the caudal periaqueductal gray (cPAG) is increased by either spontaneous morphine withdrawal or treatment with the opioid antagonist naloxone in rats [26]. In addition, the overexpression of PPE mRNA in the cPAG returned to the control levels after disappearance of morphine withdrawal signs [26]. Furthermore, increased expression of the neuronal transcription factor Fos, as a marker for neuronal activation, has been observed in the lateral, ventrolateral, and importantly, caudal vlPAG neurons during naloxone-precipitated withdrawal in rats chronically treated with morphine [23]. Fos and Jun reportedly regulate the expression of PPE mRNA [27]. Corroborating these findings, morphine withdrawal precipitates increased Fos-like immunoreactivity in the PAG [23]. These results suggest that increased Fos facilitates the synthesis of PPE mRNA during morphine withdrawal [28]. The selective anatomical localization of changes in PPE gene expression in the PAG associated with morphine withdrawal, rather than the striatum, caudate-putamen, or paraventricular-hypothalamic nucleus, is further evidence that the PAG is intimately and mechanistically linked with opioid withdrawal responses [26]. Furthermore, local administration of an enkephalin analog or peptidase inhibitors into the cPAG suppresses morphine withdrawal signs [12, 28]. Together, these findings suggest that enkephalinergic neurons in the PAG may play a critical role in the recovery phase of morphine withdrawal. 

### 5.2. The Role of Adenylyl Cyclase in Opioid Withdrawal

Chronic opioid use is associated with a decoupling of opioid G-protein coupled receptors. Importantly, there remains much to be learned about how opioid receptors become decoupled with chronic opioid administration and how this relates to the pathophysiology of opioid withdrawal that may precipitate drug-seeking behaviors and perpetuate physical dependence. The mechanism appears to involve G-protein coupled receptor-induced activation of adenylyl cyclase via protein kinase A [30, 31]. Similarly, biochemical measures of rebound, such as increased neuronal adenylyl cyclase activity, have been widely reported during opioid withdrawal [32]. Although opioid agonists acutely inhibit adenylyl cyclase activity in the PAG [33], there is a compensatory increase in adenylyl cyclase signaling during chronic treatment with morphine resulting in rebound hyperactivity of this cascade during withdrawal [32]. The mechanism is suggested by Ingram et al., who showed that opioid dependence induces efficacious coupling of mu-receptors to presynaptic inhibition in GABAergic nerve terminals via adenylyl cyclase- and protein kinase A-dependent processes in the PAG [5]. Opioid withdrawal may therefore result in a loss of this coupling and neuronal hyperexcitation of opioid-sensitive PAG neurons [25, 34].

### 5.3. A Role for Protein Kinase A in Opioid Withdrawal

Protein kinase A (PKA) refers to a family of enzymes whose activity is dependent on cellular levels of cyclic AMP (cAMP). Chronic morphine treatment increases the level of adenylyl cyclase and cAMP-dependent PKA [35]. Downstream of PKA, the expression and phosphorylation of the cAMP-regulated transcription factor cAMP response element-binding protein (CREB) are selectively upregulated after chronic morphine treatment [35]. Additional evidence for an important role for PKA on opioid withdrawal was presented by Punch and colleague who demonstrated that intra-PAG infusion of a PKA inhibitor, Rp-cAMPS, attenuates withdrawal signs in morphine-dependent rats, while a PKA activator, Sp-cAMPS, induces withdrawal-like signs in morphine naïve rats [36]. Microinjections of PKA inhibitors 1-(5-isoquinolinylsulfonyl)-2-methylpiperazine and H7 into the PAG attenuate a spectrum of opioid-withdrawal behaviors [37]. Electrophysiological and biochemical findings have suggested a role for the cAMP system in the chronic actions of opiates in PAG regions [38]. These data provide further direct evidence for the involvement of the cAMP-PKA system in the PAG in the pathophysiologic and complex behavioral manifestations of opiate withdrawal [36]. 

### 5.4. A Functional Role for PAG GABAergic Neurons in Opioid Withdrawal

Activation of a subpopulation of GABAergic neurons in the PAG also plays an important role in regulating opioid responses and withdrawal [39]. During opioid withdrawal *in vitro*, GABAergic neurons show hyperexcitability [25, 34] and increased release of GABA, but not glutamate, is observed in the PAG [5]. Hyperexcitation of GABAergic PAG neurons and the resulting increase in GABAergic inhibition of PAG output neurons toward targets in the hypothalamus and ventral medulla has been implicated in the initiation of PAG-mediated signs of opioid withdrawal [25, 40]. Bagley and colleagues found that hyperexcitation of PAG cell bodies is a result of increased GABA transporter 1 (GAT-1) currents via a PKA-dependent mechanism [34]. As noted, the upregulation of adenylyl cyclase and PKA signaling has consistently been associated with opioid withdrawal [40]. Importantly, this enhanced adenylyl cyclase signaling following chronic morphine treatment is associated with GABAergic terminal hyperexcitability during withdrawal, and this response is inhibited by a concomitant increase in endogenous adenosine in the PAG neurons [41]. GAT-1 currents could therefore contribute to the modulation of GABA release and initiation of opioid withdrawal. GAT-1 activity directly produces opioid withdrawal signs through direct hyperexcitation of GABAergic PAG neurons and nerve terminals, which presumably enhances GABAergic inhibition of PAG output neurons [42]. 

## 6. A Critical Role for Glial Activation in Morphine Withdrawal

Research on glial cells has come of age. Until a few years ago, glia cells were simply considered the glue that holds the neuronal cells together, but otherwise had no active role [43]. Abundant recent evidence confirms that glial cells are highly complex and engaged in a plethora of functions. The role of glia in, among other examples, synapse formation, synapse maturation, and plasticity and the rapid conduction of action potentials, as well as their immunological functions in the nervous system, have by now been unequivocally established [43]. Glial cells are generally distinguished in subclasses based on their diverse morphology and function. These include microglia, the immunocompetent and specialized brain macrophages; astrocytes, which represent the major glial component in the CNS and constitute up to 20–50% of the brain volume; NG2-glia, a peculiar type of glial cells that receive direct synaptic input from neurons; Schwann cells and oligodendrocytes form layers of myelin around neuronal axons in the peripheral and central nervous system, respectively [44, 45]. 

Nonetheless, our understanding of the role of glial cells in the complex syndrome of opioid dependence and withdrawal is still in its infancy. Glial cells, particularly astrocytes, envelop neuronal synapses and participate in the physiological control of synaptic transmission and plasticity via the release of synaptically effective mediators, a process called gliotransmission [29, 46, 47]. Evidence has now shown that glial cells may be critically involved in morphine dependence/withdrawal [6, 48, 49]. Morphine withdrawal induces glial activation and proinflammatory mediator expression in the different sites of the brain [50]. Chronic morphine treatment causes glial activation in the spinal cord, posterior cingulate cortex, hippocampus, and PAG [6, 51].

Chronic administration of systemic or intrathecal morphine activates spinal glia cells leading to an upregulation of proinflammatory cytokine release [52, 53]. Anti-inflammatory cytokines block the chronic morphine withdrawal-induced symptoms including pain at the spinal level [48, 52, 54]. AV411 is a blood-brain barrier permeable nonspecific phosphodiesterase inhibitor that is also known to suppress glial cell activation [49]. Systemic AV411 documents suppression of proinflammatory responses *in vitro* and *in vivo *[49]. Ledeboer and colleagues have demonstrated that coadministration of morphine with systemic AV411 suppresses morphine withdrawal and that AV411 also reduces systemic morphine-upregulated astrocytic and microglial activation markers in the brain and spinal cord [49, 55]. Coadministration of AV411 with morphine significantly reduces the naloxone precipitated opioid withdrawal behaviors across a 60 min postnaloxone time course [49, 55]. AV411 further downregulates morphine withdrawal-induced elevations of astrocytic GFAP and microglial CD11b activation markers, IL-1*β*, MCP-1, and MIP-3*α* in the PAG [50]. AV411 also prevents spontaneous morphine and oxycodone withdrawal-induced weight loss [50]. 

Although it was long assumed that opioid-induced neuroinflammation must be mediated via activation of classic opioid receptors, recent data contests this assumption [56, 57]. The series of multidisciplinary studies provided converging lines of evidence that morphine binds to an accessory protein of glial toll-like receptor 4 (TLR4), myeloid differentiation protein 2 (MD-2), thereby inducing TLR4 oligomerization and triggering proinflammation [58]. Direct activation of glial TLR4 induces overexpression of TNF*α* [59, 60]. TNF*α* is one of a handful of identified gliotransmitters [46, 61]. TLR4 inhibitor attenuated precipitated withdrawal in these morphine-dependent rats [62]. Our studies further demonstrate that morphine withdrawal induces astrocytic activation to release TNF*α* in the PAG and that, interestingly, exogenous TNF*α* injection into the PAG evokes morphine withdrawal-like behaviors [6].Thus, available evidence suggests TNF*α* plays a central role in the glial-neuronal interactions that influence drug abuse [29] by modulating synaptic transmission [47, 63].

## 7. Glia-Neuron Interactions Associated with Morphine Withdrawal in the PAG

In recent years, a considerable body of evidence has demonstrated the existence of reciprocal communication between the glial and neuronal cells, showing that the glial cells have an essential role in the regulation of the functional activity of the nervous system [64]. The glial cells release several substances that act as gliotransmitters and may influence glia-neuron communications as well as neuronal differentiation and plasticity [64]. At the neuronal interface, astrocytes exert a number of homeostatic functions that collectively contribute to maintain the microenvironment in conditions assuring optimal neuronal functions (for review see [45]). It has been estimated that the territory of each rodent astrocyte may contain 100,000 synapses and hundreds of dendrites [65, 66]. Astrocytes have privileged access to synapses. Because of the reciprocal signaling that can occur between astrocytes and synaptic terminals, these structures have been termed the “Tripartite Synapse” [67]. Astrocytes play a variety of roles in the regulation of synaptic transmission of neurons [29]. In addition to such supportive functions, astrocytes release chemical transmitters that modulate neuronal function [29]. Astrocytes respond to neuronal activity and neurotransmitters through the activation of neuronal receptors [68].

Further evidence suggests that glial cells are intimately involved in the active control of neuronal activity [67, 69]. Previous studies demonstrate a close interaction specifically between astrocytes and neurons treated with opioids [70]. Activation of astrocytes induces the synthesis and release of substances (e.g., cytokines, glutamates) capable of modulating the functions of surrounding cells, including neurons [71]. Proinflammatory mediators can exert direct actions on neurons [72] as well as induce various downstream changes that alter neuronal functions, potentially contributing to opioid withdrawal [73]. In particular, the increased release of CNS cytokines by activated glia may greatly influence or modulate neuronal functions [74, 75]. For example, TNF*α* increases the activity of glutamatergic AMPA receptors [76], and potentiates inward currents in neuronal tetrodotoxin-resistant sodium channels [77]. TNF*α* also increases spontaneous and evoked neurotransmitter release from presynaptic terminals in the hippocampus [78]. In addition, TNF*α* increases the neuronal cell surface expression of both neuronal AMPA and NMDA receptors [71, 79]. This pattern of changes is expected to create an overall increase in neuronal excitatory tone [71, 80]. Chronic adaptive molecular mechanisms involve gene expression and/or some protein kinases, which are relevant for signaling processes involving protein phosphorylation and gene expression [81]. In our recent studies, glial-neuronal interactions mediated through TNF*α* release by activated glial cells, altered neuronal mitogen-activated protein kinase (MAPK) and transcriptional factors in the PAG associated with morphine withdrawal [6].

On a subcellular level, the extracellular signal-regulated kinases (ERK1/2) is a family of serine/threonine protein kinases that have been functionally linked to addiction through phosphorylation of transcription factors leading to changes in target gene expression [81]. Recently, several studies have shown that the spinal ERK pathway contributes to naloxone-precipitated withdrawal in morphine-dependent rats [82–85]. Our studies demonstrate that phosphorylation of ERK1/2 in the PAG is upregulated in the rats with morphine withdrawal. Phosphorylation of ERK is one of the major pathways for induction of Fos. Morphine withdrawal-induced increases in ERK activity results in an enhancement in Fos [85]. The strong induction of Fos expression in the PAG was found after naloxone injection to morphine-dependent rats [23]. Morphine withdrawal-induced overexpression of Fos has been colocalized with the phosphorylation of ERK in the vlPAG [6]. CREB has also been implicated in neural plasticity, including the changes that occur during stress and drug addiction [35, 86, 87]. Chronic morphine increases levels of CREB in the CNS [88, 89]. Chronic morphine dependence exerts long-lasting effects of morphine dependence on gene expression in different sites at the supraspinal level [90, 91]. We observe an increase in PAG neuronal CREB associated with morphine withdrawal rats [6].

Evidence shows that increased TNF*α* in states of inflammation, induces pERK phosphorylation in neurons [92], and that enhanced ERK signaling facilitates Fos expression in drug abuse [93]. CREB can be activated by kinases including ERK1/2 and PKA, which induce its transcriptional activity [94]. We have found that microinjection of recombinant TNF*α* into the vlPAG induces morphine withdrawal-like behavioral response and phosphorylation of ERK1/2 and CREB, and expression of Fos in the PAG [6]. Naloxone-precipitated morphine withdrawal induces upregulation of GFAP and TNF*α* in the PAG [6]. We inject HSV vector overexpressing p55TNF soluble receptor into the PAG in morphine withdrawal rats to prevent TNF from binding TNFR on the neurons and find that TNF soluble receptor mediated by HSV vectors suppresses morphine withdrawal behaviors and phosphorylation of neuronal ERK1/2 and transcription factors of neuronal CREB and Fos [6]. In addition, TNF*α* is colocalized with GFAP, but the TNF receptor colocalizes with NeuN, suggesting that TNF*α* is derived from astrocytes and TNF receptors are primarily on the surrounding neurons. Therefore, our studies demonstrate the importance of glial-neuronal interactions in morphine withdrawal within the PAG, suggesting that glial TNF*α* binds neuronal TNF receptors to induce phosphorylation of ERK1/2 and CREB, altering the expression of neuronal activation marker, Fos. 

## 8. Summary

**Figure 1:**
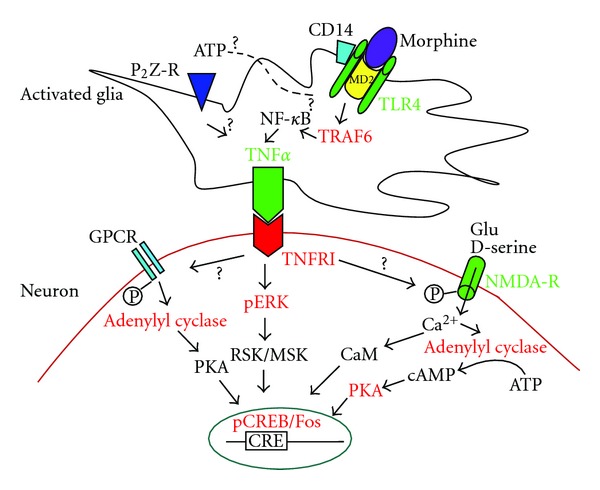
Possible cellular/molecular mechanisms of glia-to-neuron interaction in the PAG in morphine withdrawal. Morphine binds to MD-2 and induces TLR4 oligomerization on the glial cell network to induce synthesis of TNF*α* through possible TRAF6 and NF-*κ*B pathways, and so forth. Glial TNF*α* binds the TNFRI on the neurons to induce the phosphorylation of ERK, further the phosphorylation of CREB. TNFRI signal may induce phosphorylation (P) of NMDA receptor to increase Ca^2+^ influx. This increase in intracellular Ca^2+^ leads to several downstream effects including activation of CaM, adenylyl cyclase, cAMP, and further activation of PKA and changes of gene expression (CREB and Fos). GPCR may be activated to induce adenylyl cyclase and PKA activation and to increase gene transcription (CREB and Fos). GPCR: G-protein coupled receptor; glu: glutamate; CaM: calmodulin; NMDA-R: N-methyl-D-aspartate receptor.

In summary, the activation of glial cells in the PAG in response to opioids and other stimuli including inflammation, ischemia, and invading pathogens can have a profound impact on the functioning of nearby neuronal cells. Importantly, recent data show that chronic opioid exposure induces glial activation and proinflammatory mediator expression in the PAG associated with the complex syndrome of opioid dependence and withdrawal. Specifically, increased TNF*α* on glial cells activated by opioids directly impacts PAG neuronal functioning including phosphorylation of ERK1/2 and CREB, alteration in neuronal gene expression, and the precipitation of withdrawal symptoms after naloxone or opioid discontinuation. Inhibiting the biofunctions of TNF*α* suppresses chronic morphine withdrawal and reverses the neurochemical response [48, 52, 54]. Therefore, we believe that the interactions of glial-neuronal interactions mediated by the interactions of PAG glial TNF*α* with neuronal TNFR may play an important role in complex syndrome of opioid withdrawal (Figure 1). Collectively, these findings lay the groundwork for future studies aimed at further integrating the observed glial molecules and changes of neuronal markers in the PAG. These findings also suggest that the inhibition of TNF*α* may represent a targeted new approach to preventing opioid withdrawal.
